# Anatomical Consideration of the Anterolateral Ligament of the Knee

**DOI:** 10.1155/2019/5740473

**Published:** 2019-04-11

**Authors:** Ho-Jung Cho, Dai-Soon Kwak

**Affiliations:** Catholic Institute for Applied Anatomy/Department of Anatomy, College of Medicine, The Catholic University of Korea, Seoul 06591, Republic of Korea

## Abstract

Many researchers have studied the structures of the anterolateral part of the knee. Several researchers have investigated the existence of the anterolateral ligament (ALL) and its frequency has been inconsistently reported. Therefore, we assessed whether the ALL is the anatomical true ligament and studied the morphological variations of this structure. Sixty-four Korean adult cadavers (120 knees, mean age: 79.1 years) were used for this study. The lateral part of the knee joint was carefully dissected with internal rotation of the tibia. We checked the existence and morphological features and measured the dimensions (length, width, and thickness) of the ALL. The ALL was clearly distinguished from the capsulo-osseous layer of the iliotibial tract and runs obliquely from the lateral femoral epicondyle to the tibial plateau. The ALL was found in 42.5% of the samples, and 15 cadavers had ALLs in both knees. There was no prevalence difference between females and males. Most of the anterior border of the ALL was blended with the knee capsule. Therefore, we concluded that this structure is a local thickening of the capsule in the anterolateral region of the knee, where it possibly developed against some external physical stress. Therefore, the ALLs in this present study can be defined as a capsular ligament of the knee and, as per the nomenclature of the capsular ligament, can be also called the ‘anterolateral (capsular) ligament'.

## 1. Introduction

In 1879, Dr. Paul Segond described ‘a pearly, resistant, fibrous band which invariably showed extreme amounts of tension during forced internal rotation (of the knee)' [1]. He suggested that this structure exists in the anterolateral region of the tibia and is related to avulsion fracture pattern in the tibia's anterolateral proximal aspect. This anterolateral structure has been called various names including short lateral ligament [2], (mid-third) lateral capsular ligament [3], capsulo-osseous layers of the iliotibial tract (ITT) [4, 5], anterior band of the lateral collateral ligament (LCL) [6], and anterior oblique band [7]. Also, it may be referred to as the anterolateral ligament (ALL).

In 2013, Claes reported that the ALL is a distinct ligamentous structure that can be separated from surrounding structures [8]. Since then, the ALL began receiving attention as a new ligament of the anterolateral region of the knee. Many researchers have conducted morphometric studies analysing its existence and location and measuring dimensions of length, thickness, and width. However, some authors [9–12] have reported that they could not find ‘a distinct ligamentous structure' that stands out in the anterolateral part of the knee. They only described the location of capsular thickening at the anterolateral part of the knee where the ALL has been claimed to be found by other authors. Researchers mentioned that the reason for conflicting results among ALL studies is likely due to the different characteristics between fresh-frozen and embalmed cadavers and the variation in dissection methods used [12, 13]. The ALL was then studied on foetal knees. However, two differing results were obtained as one author claimed that the ALL was present in 100% of foetal specimens [14], while the other author reported that they could not locate any examples in the specimens [15]. Since there has been disagreement regarding the ALL, several scientists and clinicians developed a consensus on the anatomy of the anterolateral complex. They explained the ALL is a capsular structure within Seebacher Layer 3 located in the anterolateral part of the knee [16].

However, among these controversies, there was lack of the study about the ALL from the Asian specimens. Moreover, no cadaveric studies have been performed on a Korean population. Therefore, the main objective of the present study was to determine the anatomical features, incidence, and bilateral existence of the ALL, to compare differences between sexes using Korean fresh-frozen cadavers and to compare our findings with those of previous studies. In addition, this study sought to clearly define whether the ALL of Koreans is a capsular structure through dissection using an operating microscope.

The hypotheses of this study were that ALLs would exist in a Korean population, and the ALL would be a capsular structure in the anterolateral capsule.

## 2. Materials and Methods

In this study, 64 Korean adult fresh-frozen cadavers were used, and a total of 120 knees were dissected (female: 60, male: 60, right: 62, left: 58). Among these, paired knees were used in 56 cadavers. The mean height was 158.4 cm (female: 152.2 cm, male: 164.6 cm), mean weight was 50.8 kg (female: 45.2 kg, male: 56.4 kg), and mean age was 79.1 years (female: 84.1 years, male: 74.2 years). The specimens were excluded in two phases. First, we excluded specimens that had incision scars indicating a surgical history before dissection. Secondly, specimens with severe arthritis, osteophytes, or degeneration that could cause problems of the joint capsule and difficulty with movement were excluded on dissection.

**Figure 1:**
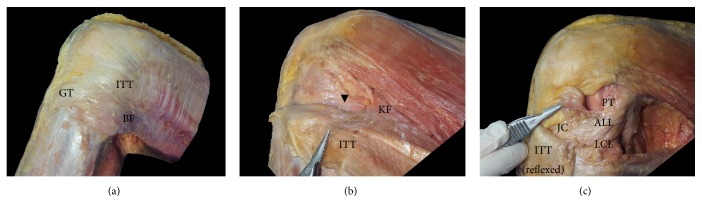
Dissection process of the ALL. (a) After removal of the skin and subcutaneous tissue, (b) after the ITT is separated posteriorly from the vastus lateralis, the Kaplan fibres and capsulo-osseous layer (Black arrow head) become evident, and (c) after the ITT including the capsulo-osseous layer is reflexed to Gerdy's tubercle, the ALL is visible within the level of the Seebacher Layer 3. ALL: anterolateral ligament, BF: biceps femoris, GT: Gerdy's tubercle, ITT: iliotibial tract, JC: joint capsule, KF: Kaplan fibres, LCL: lateral collateral ligament, LFE: lateral femoral epicondyle, LM: lateral meniscus, and PT: popliteus tendon.

For dissection, three circumferential incisions were performed at the mid-point of the thigh, knee joint line, and mid-point of the leg. A sagittal midline incision was created on the anterior part of the knee. Skin and the subcutaneous tissue were removed through these incision points. The ITT and vastus lateralis were separated using the dissection method by Lobenhoffer et al. [17], then the ITT was reflexed from the proximal part of the thigh towards its insertion after carefully detaching it from the linea aspera. The deepest layer of ITT, which called the capsulo-osseous layer, was continuous with gastrocnemius fascia, and it was detached carefully. When the LCL was palpated, the leg was internally rotated with flexed knee, and we observed if there was dense fibrous tissue (ALL) that ran superficial to the LCL in the anterolateral capsule of the knee (Figure 1). If the ALL was located, its origin and insertion were documented, and its length on knee extension and width and thickness at the joint line were measured using digital callipers (ABS Digimatic callipers, Mitutoyo). Several ALLs were observed using an operating microscope (OPMI pico, Carl Zeiss) in order to more accurately distinguish the structure.

To compare the morphometric parameters of the ALL as well as the incidence between female and male, the Student t-test and Chi-squared tests were used.

## 3. Results

The ALL could be observed at the anterolateral part of the knee joint capsule after the capsulo-osseous layer of the ITT was reflexed. The ALL appeared to be a white dense thickening structure compared to other parts of the knee joint capsule. It could be distinguished without microscope and by palpation. It was also more easily discernible when the leg was internally rotated. The ALL was located in 51 out of 120 knee capsules (42.5%) in the Korean fresh cadaveric specimens. Also, fifteen cadavers had the ALL in both knees within 56 paired cadavers (26.8%). ALLs were found in 50.0% of right knees (31/62) and 34.5% of left knees (20/58). There were no significant differences between the right and left knees (*p* = 0.09). Among the 51 ALLs, 25 were found in females (49.0%) and 26 were found in males (51.0%), suggesting no difference between sexes in the incidence (*p* = 0.58) (Table 1).

**Figure 2:**
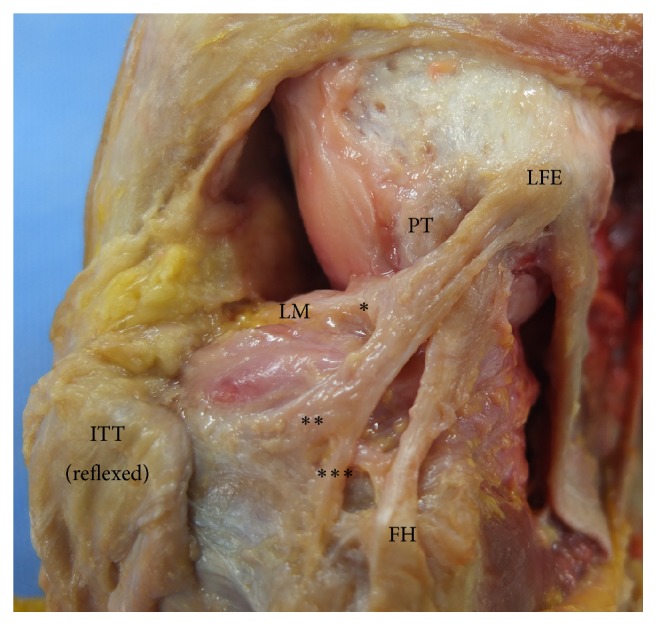
The origin and insertion of the ALL. *∗*Bundles attached to the meniscus, *∗∗*bundles attached to the tibial plateau, and *∗∗∗*bundles attached to the crural fascia. FH: femoral head, ITT: iliotibial tract, LFE: lateral femoral epicondyle, LM: lateral meniscus, and PT: popliteus tendon.

The ALL originated in the region of the lateral femoral epicondyle, which was located posterior to the popliteus tendon, and ran obliquely to insert into the tibial plateau between Gerdy's tubercle of the tibia and the fibular head. Some fibres were also attached to the lateral meniscus and crural fascia (Figure 2).

**Figure 3:**
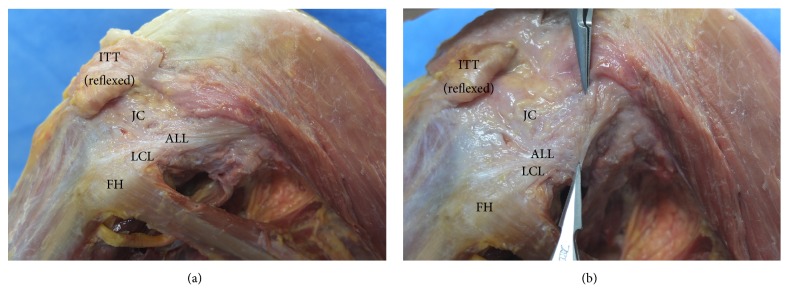
The relationship between the ALL and anterior capsule. ALL: anterolateral ligament, FH: femoral head, ITT: iliotibial tract, JC: joint capsule, and LCL: lateral collateral ligament.

The mean distance between the ALL insertion and the tip of the fibular head was 15.8 ± 4.3 mm. Among the 51 knees with the ALL, in 45 specimens (88.2%), the anterior border of the ALL was intertwined with the knee capsule, and it was difficult to clearly separate the anterior border from the capsule (Figure 3).

When the knee was extended, the mean length of the ALL was 32.4 ± 6.8 mm in total, 33.0 ± 6.7mm for females, and 31.1 ± 7.4mm for males. The width at the joint line was 5.2 ± 2.0 mm in total, 5.3 ± 2.2mm for females, and 5.1 ± 1.6mm for males. The thickness at the joint line was 1.3 ± 0.7 mm in total, 1.2 ± 0.7mm for females, and 1.5 ± 0.7mm for males. There were no sex differences in any of the comparisons (Table 2).

## 4. Discussion

One of the most important findings of this study was that the existence of the ALL was 42.5% in Korean, and we investigated morphological features of the structure. Before this study, there was a lack of the research about the ALL in an Asian population, despite considerable research on the ALL in other populations. Another important finding was that the ALL is a capsular ligamentous structure in the anterolateral capsule of the knee.

After the study by Claes et al. [8], the ALL began to attract research attention, and morphometric studies were widely conducted. However, the results of these studies were controversial. Some authors described the incidence of ALL as 100% [18–21], while other authors found it to be present in only some of their specimens [8, 22–26]. Meanwhile, there were also studies that were unable to demonstrate a ligamentous structure like the ALL in any of their specimens [9–11]. In these studies, the authors stated that they found capsular thickening or the middle third of the lateral ligament that was described by Hughston [3], but could not identify any of the distinct ligaments described by previous authors who concluded the presence of the ALL. Recently, the ALL was concluded to be a distinguishable ligament in the anterolateral part of the knee [13, 16, 27]. However, there was lack of research on the ALL in Asians. Therefore, we conducted this morphometric study to analyse the ligamentous structure in the anterolateral region of the knee using Korean cadavers.

**Figure 4:**
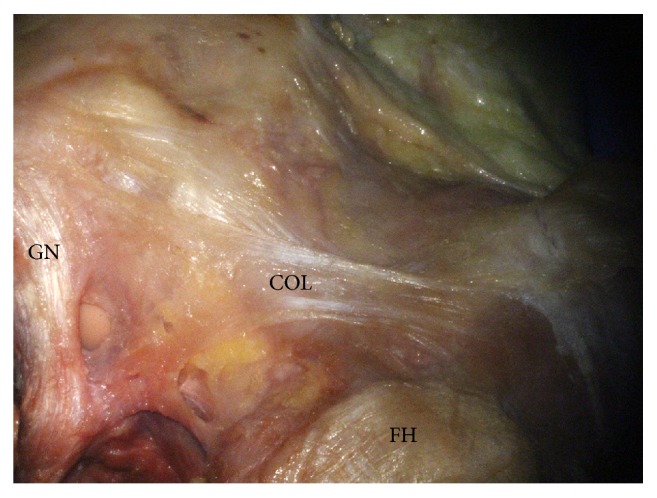
The capsulo-osseous layer of the ITT. COL: capsulo-osseous layer, FH: femoral head, and GN: gastrocnemius tendon.

The ALL of this study was clearly distinguished from the capsulo-osseous layer of the ITT (Figure 4). The capsulo-osseous layer was connected with the fascia of the lateral gastrocnemius fascia similar with the previous studies [4, 12]. Seebacher described that the lateral aspect of the knee has three distinct layers [28], and Getgood reported that the ALL is within the Seebacher Layer 3 [16]. Through the dissection of the anterolateral part of the Korean knees as layer by layer, we also agreed with the study of Getgood et al. Also, in previous studies, the ALL is usually described as being attached to the lateral femoral epicondyle proximally and to the tibial plateau distally, with some fibres inserting into the lateral meniscus. Likewise, the location was similar to that of the ALL described in previous studies.

There were many anatomical quantitative studies of the ALL. Therefore, among those, we chose some of the researches to compare with this study. In comparison to the dimensions of the ALL with the previous studies, the ALL of our study had the shortest length during knee extension. Dodds (2014) reported the longest length measurement of 59.0 mm [22], which was significantly different from the results of the present study (Table 3). The width of the ALL of our study was comparable to the width of the ALL from previous studies, and the thickness of the ALL of this study was thinner compared to that of the previous studies. We stipulated that the differences in dimensions could be attributed to the varied population groups among the studies.

**Figure 5:**
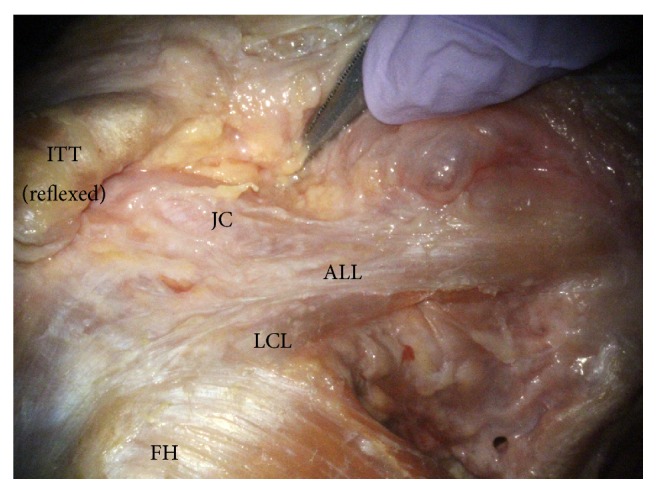
The image of intertwined fibres of the ALL and the anterior joint capsule through an operating microscope. ALL: anterolateral ligament, FH: femoral head, ITT: iliotibial tract, JC: joint capsule, and LCL: lateral collateral ligament.

In our study, we found that the fibres of the anterior border of the ALL were intertwined with the anterior capsule of the knee (Figure 5). Catherine (2015) mentioned that the ALL can be considered as capsular thickening as well as a ligamentous structure such as the glenohumeral ligament of the shoulder joint [20]. According to the author, the ‘anterolateral ligament' can be used as a synonym for the ‘capsular ligament' based on the site where it is situated. We also agree on this view, as the capsular ligament represents the local thickening of parallel bundles of collagen fibres in the fibrous capsule and is usually named after the attachment site [29]. As a result of our dissection, this thickened structure of the knee capsule was clearly distinguishable from the ITT. However, the anterior border was indistinct and was combined with the anterior joint capsule. In addition, the meniscal fibre of the ALL seemed to resemble the coronary ligament. Therefore, we concluded that this structure is a local thickening of the capsule in the anterolateral region of the knee, where it possibly developed against some external physical stress. The ALL of the present study can be defined as a capsular ligament of the knee and, as per the nomenclature of the capsular ligament, can also be called the ‘anterolateral (capsular) ligament'.

In relation to the rate of incidence, the ALLs in the present study were found in 25 female and 26 male specimens. Therefore, sex differences were irrelevant to the presence the ALL. There were significant differences in height and weight between females and males; however, we did not find any significant differences in the dimensions of ALLs between sexes. This result was different from those of a previous study by Daggett et al., which reported a significant difference between sexes in ALL thickness and length [30]. In this study, only 15 cadavers had the ALL in both knees (15/56), therefore, it showed low incidence of the ALL in both knees. Also, The ALLs were observed more in right knees (31/62 specimens) than left knees (20/58 specimens) in this study. Therefore, ALLs were not found in right and left knees symmetrically, but there was no statistical difference.

In the present study, the incidence of the ALL in Koreans was 42.5%. According to Watanabe (2016) [25], the ALL was found in 37.2% of the Japanese population. The author described that Japanese have lower frequency of the ALL compared to the Western population. In addition, the incidence of Segond fracture, which is related to the ALL, was lower in Asian populations compared to the West [25]. When we compared our result with previous studies, the ALL seemed to be prevalent in Western populations, but the results were not consistent (Table 3). According to the result of Potu's (2016) paper, the ALL was only found in only 4.16% of the Caucasian population [23]. Runer (2016) studied the ALL in the Austrian population, and the incidence was 45.5%, which is similar to the result of the present study [24]. Therefore, it appears that the population or ethnicity has little effect on the difference in incidence of the ALL among studies. It could be different by the dissection technique as mentioned by previous researcher [12].

## 5. Conclusions

The present study was conducted to confirm the presence of the ALL in the anterolateral region of the knee capsule and to analyse its anatomical characteristics using Korean adult cadavers. Capsular thickening was found in 42.5% of Korean cadavers, and this structure was separable from the ITT. Also, when the ALL was present in 88.2% of cases, the anterior border was rather indistinct and blended with the anterior part of the knee capsule. Therefore, this capsular thickening which has been controversial with its naming can be called the ‘anterolateral (capsular) ligament' with regard to its anatomical features.

## Figures and Tables

**Table 1:** Prevalence of the anterolateral ligament from Koreans.

Total (120)	Right (62)	Left (58)	Female (60)	Male (60)
42.5% (51/120)	50.0% (31/62)	34.5% (20/58)	49.0% (25/60)	51.0% (26/60)

**Table 2:** Anatomical measurements of the anterolateral ligament in Koreans (unit: mm).

	Female	Male	Total	*p*-value (Student t-test)
Length (knee extension)	33.0 ± 6.7	31.1 ± 7.4	32.4 ± 6.8	0.58
Width (at the joint line)	5.3 ± 2.2	5.1 ± 1.6	5.2 ± 2.0	0.85
Thickness (at the joint line)	1.2 ± 0.7	1.5 ± 0.7	1.3 ± 0.7	0.18

**Table 3:** Prevalence and anatomical measurements of the anterolateral ligament.

Author (year)	Subject (n)	Population (mean age)	Prevalence (%)	Length (mm)	Width (mm)	Thickness (mm)
This study (2018)	Fresh cadavers (120)	Korean (79.1)	42.5	30.1 ± 2.1 (Extension)	5.2 ± 1.5mm (Joint line)	1.0 ± 0.6mm (Joint line)
Vincent et al. (2012)	Fresh cadavers (10)	French (85.3)	100	34.1 ± 3.4	8.2 ± 1.5	2–3
Claes et al. (2013)	Embalmed cadavers (41)	Belgian (79)	97	38.5 ± 6.1 (Extension)	6.7 ± 3.0 (Joint line)	1.3 ± 0.6 (Joint line)
Helito et al. (2013)	Cadavers (20)	Brazilian (61.5)	100	37.3 ± 4.0	7.4 ± 1.7	2.7 ± 0.6
Dodds et al. (2014)	Fresh cadavers (40)	British (75)	83	59.0 ± 4.0	6.0 ± 1.0	
Caterine et al. (2015)	Fresh cadavers (19)	Canadian (70)	100	40.3 ± 6.2 (Extension)	5.1 ± 1.8 (above meniscus) 8.9 ± 2.5 (below meniscus)	1.4 ± 0.6
Kennedy et al. (2015)	Fresh cadavers (15)	American (58.2)	100	36.8 (Extension)		
Potu et al. (2016)	Formalin–fixed cadavers (24)	Caucasian (-)	4.16	34.23 (Extension)	4.04 (Joint line)	1.78 (Joint line)
Runer et al. (2016)	Embalmed cadavers (44)	Austrian (78.1)	45.5	42.2 ± 6.2 (Extension)	5.6 ± 1.3 (Joint line)	1.2 ± 0.3 (Joint line)
Roessler et al. (2016)	Fresh cadavers (20)	German (79.4)	60	39.63 ± 0.78 (Extension)	5.28 ± 0.33 (midportion)	1.52 ± 0.31 (midportion)

## Data Availability

The measurement data used to support the findings of this study are included within the supplementary information file.

## Supplementary Materials

Supplementary Materials The data of the prevalence and anatomical measurements of the anterolateral ligament in Korean attached as Supplementary Materials.
